# Assessing Prenatal and Neonatal Gonadal Steroid Exposure for Studies of Human Development: Methodological and Theoretical Challenges

**DOI:** 10.3389/fendo.2014.00242

**Published:** 2015-01-14

**Authors:** Rebecca C. Knickmeyer, Bonnie Auyeung, Marsha L. Davenport

**Affiliations:** ^1^Department of Psychiatry, University of North Carolina at Chapel Hill, Chapel Hill, NC, USA; ^2^School of Philosophy, Psychology, and Language Sciences, University of Edinburgh, Edinburgh, UK; ^3^Department of Pediatrics, University of North Carolina at Chapel Hill, Chapel Hill, NC, USA

**Keywords:** testosterone, gonadal hormones, hypothalamic–pituitary–gonadal axis, hypogonadism, sexual differentiation, prenatal development, minipuberty

Animal models provide compelling evidence that gonadal hormones, in particular testosterone, produced in the fetal and neonatal period, have life-long effects on physical characteristics, physiological functioning, and behavior (1–3). Studies of individuals with disorders of sex determination or sexual differentiation, largely congenital adrenal hyperplasia (4), Turner syndrome (5), and Klinefelter syndrome (6) strongly suggest that early gonadal steroid exposure is important in human development as well, and effects are not limited to the reproductive system alone. However, extending this work to the broader human population has proven challenging due to inherent difficulties in measuring testosterone exposure in developing fetuses and neonates.

In addition, the design and interpretation of studies may be impacted by widespread acceptance of conceptual frameworks that are not well supported empirically. For example, many researchers presume that the free hormone hypothesis, which states that unbound hormones are more readily diffusible into tissues and thus a better measure of actual exposure, is true. However, this hypothesis has not been rigorously validated and, indeed, there is evidence for active cellular uptake of SHBG-bound testosterone and for SHBG-bound testosterone mediating steroid hormone signal transduction at the plasma membrane (7). A second example: it is generally accepted that masculinization of the human brain is primarily mediated by the androgen receptor [in contrast to rodents where the estrogen receptor plays a major role (8)], in part because chromosomal males with complete androgen insensitivity (CAIS) generally espouse a female gender identity (9). However, this is not always the case (10), and other sexually dimorphic outcomes have not been carefully assessed in CAIS.

The aim of this research topic is to gather together experimental and review papers, which address the diverse challenges in assessing prenatal and neonatal gonadal steroid exposure for studies of human development with the expectation that this will allow more critical appraisal of existing studies, identify critical research gaps, and improve the design of future studies.

In terms of matrices used for the determination of testosterone exposure, Hollier et al. (11) review umbilical cord blood and Voegtline and Granger (12) review saliva. A theme running through both articles is that pre-analytic factors (collection, transport, storage, and processing) are absolutely critical in measuring testosterone exposure. Assay types and confounding factors also require careful attention. Also in the realm of measurement, Manning et al. (13) and Honekopp (14) focus on a widely used anthropometric index of prenatal testosterone exposure, the relative lengths of the second and fourth digits (2D:4D ratio). Manning et al. (13) review the evidence in support of 2D:4D and argue that this index is particularly relevant to “challenging” conditions such as aggressive and sexual encounters, which involve both organizational and activational hormone effects. Honekopp (14) carried out a meta-analysis of the relationship between 2D:4D and a functional polymorphism in the androgen receptor gene, the number of CAG repeats. He reports no evidence for a relationship and discusses the implications of this finding. Korsoff et al. (15) discuss whether prenatal testosterone transfer occurs in females from opposite sex twin pregnancies and report that anthropometric, metabolic, and reproductive characteristics relevant to polycystic ovarian syndrome (PCOS) do not differ between females from same sex and opposite sex twin pairs. Grinspon et al. (16) discuss the advantages and limitations of old and new markers used for the functional assessment of the hypothalamic–pituitary–testicular axis in boys suspected of fetal-onset hypogonadism.

It is clear that all current means of assessing early gonadal steroid exposure have unique strengths and notable weaknesses. We would argue that any results in this field should be treated with caution until converging evidence is available from multiple methods and replication. New approaches are also urgently needed. O’Connor and Barrett (17) highlight one promising area: placental gene expression.

Several papers address conceptual issues in the field. Alexander (18) highlights the potential role of the neonatal testosterone surge or “minipuberty” in male social behavior. The minipuberty has been relatively ignored by the field following early research on non-human primates, which suggested that suppression of the postnatal surge had minimal effects on a limited range of male behavioral phenotypes (19, 20). Alexander encourages us to re-examine the potential importance of the minipuberty in sexual differentiation of the brain. Xia et al. (21) also focus on the minipuberty in an experimental article probing genetic and environmental contributors to individual variation in salivary testosterone during this period. O’Connor and Barrett (17) discuss the need to consider cross-talk between the hypothalamic–pituitary–gonadal (HPG) and the hypothalamic–pituitary–adrenal (HPA) axes. Finally, Grinspon et al. (16) provide a comprehensive review of fetal-onset hypogonadism. Because these conditions vary with regard to the level of the HPA axis affected, the testicular cell population initially impaired, and the developmental period when the condition is established, studying these disorders could produce a more detailed understanding of the role of the HPG axis in developmental programing. They also make the important point that male hypogonadism cannot be limited to hypoandrogenism. They draw attention to several other testicular secretions including insulin-like-3 (INSL3), inhibin B, and anti-Müllerian hormone (AMH). Relatively little research has investigated whether these hormones impact brain development and other phenotypes beyond the reproductive system. AMH represents a particularly interesting case in this regard as it has been observed to support the survival and differentiation of embryonic motor neurons *in vitro* (22) and may regulate the development of sexually dimorphic brain areas in male mice (23, 24). There is also one report of lowered AMH and inhibin B in boys with autism, a condition with a marked male bias (25).

In conclusion, we hope that this research topic will serve as a point of reference and source of inspiration for researchers interested in the role of prenatal and neonatal gonadal steroids in human development. Ultimately, a better understanding of how individual variation in the functioning of the HPG axis impacts later health will help us explain and treat sex-biased medical conditions.

## Author Contributions

Rebecca C. Knickmeyer drafted the manuscript. All coauthors revised the manuscript for important intellectual content, and approved the final version to be published. Rebecca C. Knickmeyer agreed to be accountable for all aspects of the work in ensuring that questions related to the accuracy or integrity of any part of the work are appropriately investigated and resolved.

## Conflict of Interest Statement

The authors declare that the research was conducted in the absence of any commercial or financial relationships that could be construed as a potential conflict of interest.
